# Non-monetary valuation using Multi-Criteria Decision Analysis: Sensitivity of additive aggregation methods to scaling and compensation assumptions

**DOI:** 10.1016/j.ecoser.2017.10.022

**Published:** 2018-02-01

**Authors:** D.M. Martin, M. Mazzotta

**Affiliations:** U.S. Environmental Protection Agency, Office of Research and Development, Atlantic Ecology Division, 27 Tarzwell Drive, Narragansett, RI 02882, USA

**Keywords:** Ecosystem services, Trade-offs, MCDA, Decision making

## Abstract

Analytical methods for Multi-Criteria Decision Analysis (MCDA) support the non-monetary valuation of ecosystem services for environmental decision making. Many published case studies transform ecosystem service outcomes into a common metric and aggregate the outcomes to set land use planning and environmental management priorities. Analysts and their stakeholder constituents should be cautioned that results may be sensitive to the methods that are chosen to perform the analysis. In this article, we investigate four common additive aggregation methods: global and local multi-attribute scaling, the analytic hierarchy process, and compromise programming. Using a hypothetical example, we explain scaling and compensation assumptions that distinguish the methods. We perform a case study application of the four methods to re-analyze a data set that was recently published in *Ecosystem Services* and demonstrate how results are sensitive to the methods.

## 1. Introduction

The incorporation of ecosystem services (ES) into environmental decision making is an important topic and motivator of current research. Much of the research on ES focuses on ecological understanding of how ecosystems provide useful goods and services, economic understanding of how those goods and services are valued, and connections between the provision of ES and social benefits. Frameworks for integrating ES into environmental decision making facilitate the screening of management alternatives where the provision of ES is a valued outcome (NRC, 2004; USEPA, 2009; Wainger and Mazzotta, 2011; Olander et al., 2017). Many of these frameworks emphasize the need to quantify and evaluate tradeoffs in the value of ES outcomes, which may rely on monetary or non-monetary valuation methods.

Monetary valuation methods result in estimates of marginal changes to ES in monetary units (e.g., dollars), while non-monetary valuation methods result in estimates of ES or their benefits, both quantitative (e.g., species saved, number of people or homes affected) and qualitative (e.g., “poor,” “good,” “excellent”). Non-monetary valuation is a way for research analysts to address the range of ES values to decision makers or other stakeholders, without excluding those that are difficult to monetize (Chan et al., 2012). Aggregating non-monetary values is less common than aggregating monetary values because it is difficult to aggregate such data, which often are not measured in common units. Yet, it is often useful to be able to aggregate a set of non-monetary measures into a single value or score that can be used to compare management alternatives for decision making purposes.

One approach to the problem of aggregation is to use mathematical concepts that have been made popular within the field of Multi-Criteria Decision Analysis (MCDA; Langemeyer et al., 2016; Saarikoski et al., 2016). Since the 1960s, over 100 methods for MCDA have been developed to support the evaluation of environmental problems with multiple competing goals, objectives, and performance measures. Regarding ES assessment, these methods can aggregate multiple potential ES measures for pre-determined management alternatives at different geographic scales, including single or multiple sites, watersheds, and planning districts, so that a clear ranking of management alternatives at those locations is achievable. Perhaps the most attractive features of these methods are their abilities to transform incommensurable data (i.e., monetary and non-monetary values) into non-monetary, dimensionless values, and to mathematically incorporate people’s preferences into the aggregation.

A new collection of MCDA research articles are using additive functions (e.g., weighted linear combination) to aggregate monetary and non-monetary ES outcomes for environmental decision making (e.g., Liu et al., 2013; Favretto et al., 2016; Wam et al., 2016). Many studies are applying what is referred to as “spatial MCDA,” where Geographic Information System mapping of ES is combined with an additive function to aggregate ES outcomes at a spatial unit (e.g., Kremer et al., 2016; Grêt-Regamey et al., 2016; Vogdrup-Schmidt et al., 2017; Tobón et al., 2017).

It is important for research analysts to recognize that different approaches to aggregating ES reflect different underlying rationales and mathematical assumptions. Results will be sensitive to those assumptions, and analysts should be transparent with decision makers about their choices and be prepared to re-evaluate their MCDA models based on input from decision makers.

In this article, we explore two important classes of assumptions, those related to scaling and compensation, and demonstrate how choice of method and its underlying assumptions can affect the ranking of management alternatives. Scaling refers to how non-monetary ES outcomes are transformed into a common metric for meaningful aggregation, whereas compensation refers to the extent to which an undesirable ES outcome will be compensated by desirable outcomes on other ES. To explain these assumptions and demonstrate their implications for decision making, we use a hypothetical example that illustrates four common additive aggregation methods: multi-attribute scaling, both global and local (Belton and Stewart, 2002; UK, 2009), the analytic hierarchy process (Saaty, 1980), and compromise programming (Zeleny, 1973). We present a case study application of the methods using a recently published data set in *Ecosystem Services* (Favretto et al., 2016) to demonstrate how results can differ among methods.

## 2. Mathematical concepts for aggregating non-monetary ecosystem service values

The case studies performed in recent articles using additive aggregation have similar problem formulations. They are designed to estimate and evaluate the overall performance of specific land management alternatives *a_i_*, each with a finite set of ES criteria *c_j_*, defined loosely as measurable and manageable contributions of ecosystem structure and function to human well-being (Burkhard et al., 2012). For each management alternative, there is a set of quantitative and qualitative ES criteria performance values *z_ij_* based on the potential ES outcomes provided at a site or spatial unit. The criteria performance values are estimated using available market information, natural and social science models or metrics, or expert opinion-based models. We assume that measurements for each of the criteria performance values do not depend on any of the other criteria performance values.

Based on these problem formulation assumptions, a benefit function *B_i_*, sometimes referred to in the literature as a value function, is used to aggregate criteria performance values into an overall non-monetary value for each alternative. Additive benefit functions are the most common; they appear as:

(1) $$B_{i}=\sum_{j=1}^{k}W_{j}\chi_{\mathrm{ij}}$$

for all criteria *j* = 1, …, *k*, alternatives *i* = 1, …, *m*.

where *B_i_* is the overall value or benefit of alternative *i*; *w_j_* are importance weights for the criteria; *χ_ij_* are criteria performance values that have been transformed based on the methods discussed in this article. In order to aggregate, it is necessary to transform each of the original criteria performance values *z_ij_*, which are often measured using different metrics and scales, into a commensurable value *χ_ij_* that can be aggregated. Criteria performance values are almost always transformed as scaled numbers in the benefit function to facilitate comparisons across criteria. Importance weights generally reflect the importance of ES criteria to relevant beneficiaries or stakeholders; they are scaled to an interval (0–1) and sum to one. By combining criteria performance values into an aggregate benefit value, Eq. (1) estimates a single overall benefit score for each management alternative, which can make it easier for decision makers to compare and rank many management alternatives.

### 2.1. Four methods to transform ecosystem service values into a common metric

Additive aggregation methods for MCDA differ in terms of how quantitative and qualitative criteria performance values *z_ij_* are transformed into commensurable performance values *χ_ij_* before being aggregated using Eq. (1). In this section, we briefly explain four common methods. The first two methods are used in multi-attribute value assessment – global and local multi-attribute scaling (Belton and Stewart, 2002; UK, 2009), hereafter referred to as global and local scaling. The second two are well-established additive aggregation methods for MCDA – the analytic hierarchy process (Saaty, 1980) and compromise programming (Zeleny, 1973).

#### 2.1.1. Global scaling

One of the most practical procedures is to transform criteria performance values using upper and lower numerical boundaries (Keeney and von Winterfeldt, 2007). Global scaling refers to transformations using the maximum and minimum possible values for each criterion as upper and lower boundaries. These boundaries are often assigned prior to actual criteria measurements for the alternatives. Quantitative performance values are transformed to a selected range, such as 0 to 100; linear transformation is commonly used:

(2) χij=Zij−Zj^|Zj^−Zj^^|∗100

where Zj^ and Zj^^ are the worst and best *possible* measurements for each criterion, respectively. Qualitative data may be assigned numbers on a constructed scale (e.g., “none” = 0, “poor” = 25, “fair” = 50, “good” = 75, “excellent” = 100) before they are transformed using Eq. (2).

With global scaling, the lowest and highest transformed performance values for most criteria will often not be 0 and 100, since the measured values will typically not encompass the worst or best possible outcomes for the criteria. Because of this, the transformed criteria performance values will span different sized ranges (e.g., one criterion may span the range of 0 to 100 while another may only span the range of 40 to 60). This difference in range makes the global scaling method subject to individual criteria having greater influence on the results because criteria with larger ranges act like a weight on the results (Otway and Edwards, 1977; Section 3.1). An advantage of the global scaling method is that it allows for later addition of alternatives to the decision problem without disrupting criteria boundaries.

#### 2.1.2. Local scaling

Local scaling uses the maximum and minimum criteria performance values that are measured to set the upper and lower boundaries of the transformation. As with global scaling, linear transformation is commonly used:

(3) $$\chi_{\mathrm{ij}}=\frac{Z_{\mathrm{ij}}-Z_{j}^{*}}{\left|Z_{j}^{*}-Z_{j}^{**}\right|}*100$$

where $Z_{j}^{*}$ and $Z_{j}^{**}$ are the worst and best *actual* measurements for each criterion, respectively.

In contrast to global scaling, local scaling will always result in transformed performance values ranging from 0 to 100, where the lowest measured value for each criterion scales to 0 and the highest to 100. All criteria performance values will have equal influence on the final scores for the alternatives, assuming the criteria are weighted equally by decision makers. This feature could amplify the overall effect of criteria with smaller ranges relative to other criteria.

Many of the studies referenced in Section 1 use either the global or local scaling methods. Some articles explicitly specify the use of global scaling (Favretto et al., 2016; Wam et al., 2016) and local scaling (Tobón et al., 2017), but some don’t specify the method used (Liu et al., 2013; Kremer et al., 2016; Vogdrup-Schmidt et al., 2017). As will be demonstrated in this article, the choice of transformation technique is a critical dimension of transparency that should be discussed with decision makers and explicitly stated in publication.

#### 2.1.3. Analytic hierarchy process

The analytic hierarchy process is an alternative approach to assessing the overall value of management alternatives. This method transforms the criteria performance values of alternatives using ratio scales and eigenvalue analysis. The method is perhaps most well-known for analyzing incommensurable and even immeasurable criteria based on qualitative judgements (Saaty, 2013).

When quantitative performance values need to be transformed, we normalize the performance values of the alternatives per criterion such that the transformed performance values sum to unity:

(4) $$\chi_{\mathrm{ij}}=\frac{Z_{\mathrm{ij}}}{\sum_{i=1}^{m}Z_{\mathrm{ij}}}$$

Ratio scales are preserved in this normalization (Forman, 1993), which allows for easy calculations, making computational needs for using Eq. (1) similar to those of the global and local scaling methods.

In situations where the consistency of the criteria performance values is questioned, particularly when transforming qualitative performance values, an alternative method may be used which involves comparing the alternatives in pairs per criterion. To do this, we assume that each alternative, *a_i_*, …, *a_m_*, has an *importance value* assigned to it, *υ*_1_, …, *υ_m_*, per criterion; this is done by considering the smallest criteria performance value as the unit, and all larger performance values as multiples of that unit. The new importance values are ratio scale translations of the importance of the alternatives as they are compared in pairs. For each criterion *j*, the paired comparisons of importance values is represented as a ratio in the matrix *A_j_*, which satisfies the reciprocal property (Saaty, 1980):

(5) $$A_{j}=\begin{array}{c} a_{1}\\ \vdots \\ a_{m} \end{array}\;\left(\begin{array}{ccc} a_{1} & \cdots & a_{m}\\ \upsilon_{1}/\upsilon_{1} & \cdots & \upsilon_{1}/\upsilon_{m}\\ \vdots & \ddots & \vdots \\ \upsilon_{m}/\upsilon_{1} & \cdots & \upsilon_{m}/\upsilon_{m} \end{array}\right)$$

From this matrix, eigenvalue analysis approximates a vector of transformed criteria performance values *χ_j_* for the management alternatives that satisfies:

(6) $$A_{j}\chi_{j}=\lambda_{\max}\chi_{j}$$

where *χ_j_* is the principle right eigenvector of *A_j_* that sums to unity and is considered to be the set of transformed performance values of the alternatives for that criterion; *λ_max_* is its corresponding maximum eigenvalue. The transformed values over all criteria can then be used to calculate benefit function values using Eq. (1).

For qualitative data transformations, Saaty’s 9-point importance scale can be used to directly assign ratio scale importance values in the *A_j_* matrix for calculating *χ_j_* (Table 1; Section 4.3). The analytic hierarchy process has an established process to analyze the logical consistency of the *A_j_* matrix as qualitative judgements can often be inconsistent; an example is provided in the Supplementary material. The method also involves some theoretical assumptions that are challenged by proponents of multi-attribute value theory (Dyer, 1990; Forman and Gass, 2001; Belton and Stewart, 2002; Gass, 2005), which are not covered in this article.

#### 2.1.4. Compromise programming

Compromise programming is not commonly used in MCDA approaches to ES assessment. However, we include it as an alternative approach because it uses a simple additive aggregation technique, making computational needs similar to those of the other methods, and it is well-regarded in some of the comprehensive texts on MCDA (e.g., Belton and Stewart, 2002) and mapping-based MCDA (Malczewski and Rinner, 2015).

Unlike the global and local scaling and analytic hierarchy process methods, compromise programming does not utilize a benefit function. Instead, it is an interactive type of approach that uses geometry to estimate the “distance” of each alternative from a specified ideal outcome on all criteria. Like the other methods, it uses an additive aggregation function, referred to as a distance function *D_i_*, and an alternative linear transformation:

(7) $$D_{i}=\sum_{j=1}^{k}W_{j}^{p}\chi_{\mathrm{ij}}^{p}$$

where *p* is a distance norm;

(8) $$\chi_{\mathrm{ij}}=|\frac{Z_{\mathrm{ij}}-Z_{j}^{\#\#}}{Z_{j}^{\#}-Z_{j}^{\#\#}}|$$

where $Z_{j}^{\#}$ and $Z_{j}^{\#\#}$ are the “worst” and “ideal” measurements for each criterion across the alternatives, respectively, as determined by decision makers.

For compromise programming, it is assumed that the measured data are bound by some ideal and worst criteria values over the alternatives. The set of ideal criteria values are translated into an ideal solution point that exists at unity in geometric space (e.g., coordinate with all 100 scores).

Unlike global scaling, which uses the best possible solution as a bound, the ideal solution, where every criterion achieves its ideal value, is not real or feasible. Therefore, the scaling function (8) transforms criteria performance values by a measure of the distance between the data and the ideal performance value. The transformed performance values are then aggregated for each alternative using Eq. (7), and these distance scores are used to rank the alternatives.

Distance norm values of 1 < *p* ≤ ∞ weight deviations from the ideal point higher with greater distance; the *p* parameter controls the level of compensation. Decision makers may select *p* > 1 so that deviations from the ideal solution are penalized in proportion to their distance; Euclidean distance (*p* = 2) is commonly used. When applying the compromise programming method, the ideal solution may be amended by selecting criteria values that are less than the best possible, that is, they are good enough or satisfactory for decision making (Zeleny, 1974). This feature of the method challenges theoretical assumptions in multi-attribute value theory, which are not covered in this article.

As with the global scaling method, it is important to note that results are sensitive to how differentiated the criteria values are in the measured data set. If there is substantial variation in criteria performance value ranges, then re-scaling the data using, for example, Eq. (3) can be done prior to applying Eq. (8) to avoid certain criteria inadvertently dominating the results (Martin et al., 2017; Section 3.1).

## 3. Two critical assumptions

**Fig. 1:**
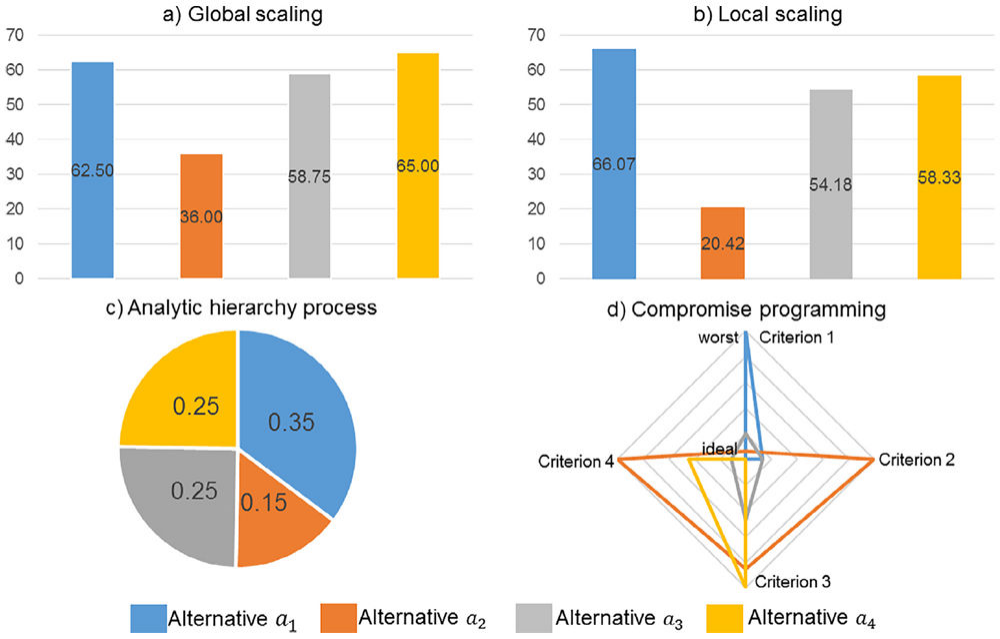
Benefit function values from the hypothetical multi-criteria problem using the global scaling (a), local scaling (b), and analytic hierarchy process (c) methods. Higher benefit function values correspond to more preferred alternatives. Distance function values using the compromise programming method (d). Performance values for each criterion range from “worst” = 1 to “ideal” = 0; alternatives closer to the ideal (lower distance function values) are preferred.

There are numerous assumptions that underlie these methods. Two classes of assumptions related to scaling and compensation are critical to the recent collection of articles using additive aggregation for ES assessment. These assumptions are important topics for research analysts to be transparent about with decision makers. We explain these assumptions using a hypothetical multi-criteria problem to evaluate four alternatives with four criteria. Data and summary statistics are provided in Table 2. Transformed data, benefit function scores, and final rankings are provided in Tables 3-6. Visualizations of the results are provided in Fig. 1. A spreadsheet with calculations for the example is available in the Supplementary material. We assigned equal importance weights to the criteria (0.25 for each criterion), which simplifies comparisons across different scaling and compensation assumptions (for references on how importance weights might impact results, see Choo et al., 1999; Steele et al., 2009).

### 3.1. Scaling

The four methods differ in terms of scaling technique performed, which can affect the ranking of alternatives. Each method has different implications for decision making. In our example, notice how the transformed performance values for Criteria *c*_2_, *c*_3_, and *c*_4_ differ between the global scaling (Table 3) and local scaling (Table 4) methods. This is due to different upper and lower boundaries used in Eqs. (2) and (3). The global scaling method ranked Alternatives *a*_4_ and *a*_1_ first and second, respectively, and the local scaling method ranked those alternatives second and first, respectively.

This occurs because the global scaling method transforms criteria performance values based on the best and worst possible outcomes for each criterion, so that performance values that do not span the full range of possible outcomes have less influence on the overall rankings as compared to the local scaling approach. The performance value measurements of Criteria *c*_2_, *c*_3_, and *c*_4_ are transformed to the same range (0–100) using the local scaling method (Table 4). Using the global scaling method (Table 3), however, Criteria *c*_2_, *c*_3_, and *c*_4_ range from 30 to 100, 10 to 75, and 25 to 100, respectively. This is important because variation in the transformed scores acts like a weight on the results (Otway and Edwards, 1977). The significance for decision making is that the global scaling method effectively ranks alternatives relative to potentially better or worse alternatives that are not in the set of evaluated choices, while the local scaling method ranks the alternatives relative only to the alternatives that are being evaluated.

Using the global scaling method, the transformed data for Criterion *c*_1_ influences the results more so than the other criteria because its range (0–100) is greater than the other criteria, regardless of the criteria weighting scheme. Consequently, Alternative *a*_4_ is preferred. If, for example, the range of criteria performance value measurements for Criteria *c*_1_ and *c*_3_ were lessened or increased, respectively (e.g., we change the minimum measured criterion values of Criteria *c*_1_ and *c*_3_ to 0.35 and 0 in Table 2, respectively), then Alternative *a*_1_ would rank first using the global scaling method while the ranking of alternatives using the other methods would remain unchanged. UK (2009) claim that there should not be a difference in the ranking of alternatives between the global and local scaling methods, but we and others (Steele et al., 2009) disagree based on our hypothetical example and case study application (Section 5).

The analytic hierarchy process method provides the same ranking of the first and last alternatives as the local scaling method, but gives equal ranks to Alternatives *a*_3_ and *a*_4_, as opposed to the local scaling method, which ranks Alternative *a*_4_ higher than Alternative *a*_3_ (Table 5). In general, the magnitude of differences in transformed performance values and benefit function values is greater using the local scaling method than using the analytic hierarchy process. This is due in part to the different normalizations of the measured data set across methods.

The analytic hierarchy process distributes value among the alternatives so that the transformed performance values for each criterion sum to unity across all alternatives evaluated. In other words, the analytic hierarchy process interprets a criterion performance value of an alternative as a reflection of its relative contribution to that criterion’s value as it compares to the other alternatives. This is mathematically and intuitively different than local scaling, which uses Eq. (3) to assign a reference to the highest criterion value and all other values proportionately less across the alternatives.

The mathematical distinction is twofold: (i) ratio scales are preserved in the transformed performance values using the analytic hierarchy process, which implies that the method explicitly compares alternatives to each other and not to a reference, and (ii) there are fewer zero transformed values using the analytic hierarchy process method because few criteria have zero value relative to others (Table 5). Using the local scaling method, each criterion will have a zero value for the lowest-valued alternative (Table 4). For these reasons, the difference between the scaled performance values and, consequently, the benefit function values may be smaller using the analytic hierarchy process method.

Intuitively, the decision maker compares the alternatives in terms of their proportional values, as in a pie chart, using the analytic hierarchy process (Fig. 1c), where the alternatives each contribute a relative proportion in value to achieving the problem goal(s). In contrast, the global and local scaling approaches require the decision maker to compare the alternatives’ overall values, as in a histogram (Fig. 1a and b), where each alternative’s value is independent of its proportional relationship to the others.

### 3.2. Compensation

In our example (Table 2), notice that Alternative *a*_3_ has performance values that are less than the “best” but higher than the “worst.” It is ranked third using the global scaling (Table 3) and local scaling (Table 4) methods and second using the analytic hierarchy process method (Table 5). However, Alternative *a*_3_ ranks first using the compromise programming method (Table 6) because its scaled values are collectively closer to the ideal solution point in geometric space (Fig. 1d). This variation in rank is based in part on the compromise programming method being less compensatory, because unit increases do not equally compensate for unit decreases when *p* > 1. In this context, decision makers need to take a position: Are alternatives with well-balanced ES values preferred to alternatives that are well valued on a number of ES but worse on others (Bouyssou, 1986)? In other words: How strongly should higher valued criteria be allowed to compensate for lower-valued criteria?

The global scaling, local scaling, and analytic hierarchy process methods are generally considered to be compensatory approaches because they focus on maximizing overall value and thus allow high values to compensate for low values, whereas the compromise programming method is less compensatory because it focuses on minimizing distance measurements and thus allows flexibility in how much low values are compensated by high values. It is important to note that, according to the scaling properties of the analytic hierarchy process (i.e., differentiation in criteria performance values; Section 3.1), an argument can be made that the analytic hierarchy process is less compensatory than the global scaling and local scaling methods in some, but not all, situations because the method distributes the value of each criterion among the alternatives. In addition, the compensatory properties of these methods can be altered by changing the weights placed on the individual criteria. In our example, we used equal weights.

## 4. Case study

We reviewed several recently published applications of MCDA for ES assessment and selected one study (Favretto et al., 2016) to use as a case study evaluation of the four approaches described in this article. Favretto et al. (2016), which appeared in a previous issue of *Ecosystem Services*, is among a limited number of articles that published their data set (Table 7). The article reported on an evaluation of ES trade-offs with the proposed implementation of four land management alternatives in the Kgalagadi District, southern Botswana: (i) communal livestock grazing, (ii) private cattle ranches, (iii) private game ranches, and (iv) wildlife management areas.

Nine monetary and non-monetary ES criteria were measured with five quantitative and nine qualitative sub-criterion indicator metrics (Table 7). Quantitative data came from relevant land management assessment reports, stakeholder interview data, and/or financial statements. Qualitative data were generated from stakeholder interviews and measures were categorized as “Very low,” “Low,” “Medium,” “High,” and “Very high,” which were translated into 0, 25, 50, 75, and 100 scores, respectively. Mean values of the indicators for each criterion and the global scaling method were used to estimate a single benefit function value for each land management alternative. An initial criteria weighting scheme was estimated from direct stakeholder input (left-hand column in Table 7). Sensitivity analyses were performed using five alternative criteria weighting schemes and one alternative scoring scheme that altered ES values.

We tested the four additive aggregation methods described in this article on the data set to note differences in rank among the methods. First, we implemented an iteration using equal weights that served as a baseline for our analysis. Second, we implemented sensitivity iterations using the weighting schemes from Favretto et al. (2016). In the following sections, we explain the data analysis assumptions that we made for applying each transformation prior to benefit or distance function calculations.

### 4.1. Global scaling

As Favretto et al. (2016) used the global scaling method, reproducing their results was straightforward. We transformed the quantitative data using Eq. (2). Implementing this step required using different maximum and minimum global endpoints for each quantitative indicator (see *Range* column in Table 7), which resulted in different ranges of transformed performance values. The qualitative data were assigned numbers using the 0/25/50/75/100 scale before transformation using Eq. (2). The initial criteria weights were equally distributed among the sub-criteria indicators for each ES.

We also reproduced the paper’s sensitivity results with the exception that we noticed the authors changed certain ES weights in the sensitivity iterations without normalizing the set of weights to sum to one. This resulted in the sensitivity weighting schemes summing to a number other than one. Nevertheless, we applied the weighting schemes used by the authors of that paper to reproduce their results. We did not perform the sensitivity iteration that altered ES values because that analysis was based on altering mean values per criterion (Table 4 in Favretto et al., 2016) instead of using the indicator values (Table 7 in this article). We checked the benefit function values based on implementing the global scaling method against the Favretto et al. (2016) results to confirm that our global scaling calculations reproduced their results precisely.

### 4.2. Local scaling

We used Eq. (3) to scale the quantitative data onto a 0 to 100 scale using each indicator’s measured end points. The qualitative data were assigned numbers using the 0/25/50/75/100 scale before transformation using Eq. (3). We used these values and each alternative weighting scheme for the indicators to calculate new benefit function values. The sensitivity analysis was performed in the same manner.

### 4.3. Analytic hierarchy process

Data analysis using the analytic hierarchy process took a few extra steps to get from the data set in Table 7 to a transformed data set for using Eq. (1). First, we performed vector normalization using Eq. (4) on the quantitative indicator data in Table 7 with the exception of the net profit of meat production indicator. That indicator ranged between negative and positive numbers. We normalized the values using Eq. (3), and then normalized those values using Eq. (4) to preserve ratio scales. Second, we performed eigenvalue analysis on the remaining nine qualitative indicators in Table 7. We set up nine reciprocal matrices using Eq. (5) for each qualitative indicator and performed pairwise comparisons of the land management alternatives using Saaty’s 9-point pairwise importance scale (Table 1). For example, if a “low” value was compared to a “low” value, it was given the ratio “1/1” denoting equal importance. Similarly, if a “low” value was compared to a “very low” value, it was given the ratio “3/1,” and if a “low” value was compared to a “medium” value, it was given the ratio “1/3.” Lastly, we approximated a vector of performance values for each reciprocal matrix using Eq. (6). Our new table of transformed indicator performance values were used to calculate benefit function values using Eq. (1).

### 4.4. Compromise programming

We transformed the quantitative data into distance measurements using Eq. (8) and assumed that the “ideal” and “worst” values were reflected in the data set. Likewise, the qualitative data were assigned numbers using the 0/25/50/75/100 scale before transformation using Eq. (8). We used those data and the alternative weighting scheme to calculate distance function values using Eq. (7) with the goal of minimizing the Euclidean distance (*p* = 2) between the management alternatives and an ideal but non-feasible alternative.

## 5. Results

As expected, our re-analysis of the Favretto et al. (2016) data set produced different results for each method. Because the overall benefit and distance function values resulted in different dimensionless scales, we did not conduct statistical comparisons. Rather, in accordance with the field of MCDA, we compared the results based on how each land management alternative ranked using the overall benefit or distance function calculations (Table 8).

Examining the baseline iteration using equal weights, the highest ranked alternatives differ across methods. Notably, the highest ranked alternative using global and local scaling was communal livestock grazing, whereas the highest ranked alternative using the analytic hierarchy process and compromise programming was wildlife management areas.

There are discernible differences in rankings between the global scaling method used by Favretto et al. (2016) and the other methods. This outcome is consistent with the fact that each of the methods use different scaling and compensation assumptions to determine overall value.

## 6. Discussion

Although Favretto et al. (2016) recognize that their results may vary with changes in performance values and weights for the ES criteria, our re-analysis shows that results also depend on the choice of MCDA technique for transforming ES performance scores into commensurable scales and aggregation. These results are due in part to the way scaling is performed on the data set and on the compensation features of the methods.

As shown in the data set (Table 7), the communal livestock grazing alternative performed better than the wildlife management areas alternative for seven out of the 14 indicators, whereas the wildlife management areas alternative performed better than the communal livestock grazing alternative for four out of the 14 indicators, all other indicator values between the two alternatives being equal. This explains why the communal livestock grazing alternative is consistently ranked higher than the wildlife management areas alternative by the global and local scaling methods (Table 8). However, the *overall* performance of the wildlife management areas alternative is high relative to the other alternatives across all criteria, which is meaningful for calculations using the analytic hierarchy process. Likewise, the wildlife management areas alternative is closer overall to an ideal but non-feasible alternative where all the indicator values are maximized, which is meaningful for the compromise programming method. For these reasons, the wildlife management areas alternative would rank as preferred over the communal livestock alternative in decision making situations where a more balanced outcome across criteria is desired, versus maximizing value over all outcomes (without the use of weights).

Rankings for the other management alternatives were somewhat variable across methods. The private game ranches alternative ranked last for most iterations for the same reasons that communal livestock grazing ranked first for most iterations. However, throughout the sensitivity iterations, the private cattle ranches and wildlife management areas alternatives differed in rank among the local scaling, analytic hierarchy process, and compromise programming methods that were not used by Favretto et al. (2016). Although the variations in ranking are due in large part to the changing criteria weights and their effect on the results, the rankings are also affected by the scaling and compensation assumptions. This has implications for how the goals of a decision problem should be incorporated into the analysis through method selection. If decision makers aim to constrain their decisions within the bounds of known alternatives, or if less compensatory, well-balanced alternatives are important to decision makers, then identifying a clear second and third ranking among the management alternatives is not straightforward.

A critical dimension of transparency that is not often considered in ES studies using MCDA concerns providing decision makers with implications of the methodological assumptions of the various MCDA techniques. Our hypothetical example and case study results point to the importance of decision makers understanding and specifying which assumptions are most relevant or desired for their particular decision context: (i) an emphasis on high-valued ES and overall benefits for each alternative (global or local scaling; Fig. 1a and b); (ii) a focus on relative ES values over all alternatives (analytic hierarchy process; Fig. 1c); or (iii) an emphasis on achieving well-balanced values across all ES (compromise programming; Fig. 1d). From this perspective, it is the decision maker or their stakeholder constituents who need to specify the preferred method based on how they prefer to scale and compensate for monetary and non-monetary values.

Research is ultimately limited in time and resources. Research analysts are tasked with ensuring the maximum level of participation from decision makers throughout the MCDA process; yet, decision makers or funding agencies may be uncomfortable or unable to give methodological input. There is a knowledge gap in how to better handle transparency about methodological assumptions with decision makers. In our experience, we have encountered both desire and indifference from decision makers in choosing methodological assumptions as we carry out an MCDA process. Regardless of choice of technique, relevant assumptions should be reported in publication.

## 7. Conclusions

In this article, we explained how scaling and compensation assumptions of four different additive aggregation methods for MCDA lead to different rankings of management alternatives. We demonstrated the sensitivity of results to common assumptions of each method through a hypothetical example and through reanalyzing a published data set. These implications of different methods have not generally been made explicit in the field of non-monetary valuation of ES, nor are they emphasized in publications. This article complements other overviews of MCDA in the literature (e.g., Langemeyer et al., 2016; Saarikoski et al., 2016), with a more in-depth description of the scaling and compensation assumptions of several aggregation techniques. In a companion study, we are expanding on this work, exploring outranking techniques (e.g., ELECTRE, PROMETHEE) using a case study where decision makers give input on methodological assumptions.

Research in this field is growing (Huang et al., 2011) and we expect to see more ES assessments that determine an overall non-monetary value of environmental management alternatives. We have shown that certain multi-attribute value functions (e.g., global and local scaling methods) produce results that differ from other additive aggregation methods that use alternative value and distance measurements. Although the different assumptions underlying the methods outlined in this article can lead to different rankings of alternatives, one method is not inherently better than others. This is why it is critical that ES analysts and decision makers jointly decide which method is a better fit for aggregating ES values in a particular decision-making context. Since decision makers may be unaware of methodological assumptions, ES analysts should be transparent about the methods they choose, to ensure that the assumptions match the objectives and preferences of decision makers. Doing this could produce results that are most meaningful to decision making. This article provides context to inform that choice.

## Supplementary Material

Sup 1

## Figures and Tables

**Table 1:** Saaty’s 9-point pairwise importance scale. Modified from Saaty (1980).

Ratio importance value scale	Judgement	Explanation
1/1	Equal importance	The two alternatives are equally important
3/1	Moderate importance of one over another	Experience and judgement slightly favors one alternative over another
5/1	Strong importance	Experience and judgement strongly favors one alternative over another
7/1	Very strong importance	An alternative value is strongly favored over another and its dominance is demonstrated in practice
9/1	Extreme importance	The evidence favoring one alternative over another is of the highest possible order of affirmation
Reciprocal example	If element *υ*_1_/*υ*_m_ has one of the above ratio scale measurements assigned to it (e.g., “3/1”), then *υ*_m_/*υ*_1_ is the reciprocal value (e.g., “1/3”)	

**Table 2:** Hypothetical multi-criteria problem. Measured data and method-relevant calculations are given to set up transformation and additive aggregation.

	Criterion *c*_1_	Criterion *c*_2_	Criterion *c*_3_	Criterion *c*_4_
Alternative *a*_1_	0	2.25	75	Excellent
Alternative *a*_2_	0.74	0.9	15	Poor
Alternative *a*_3_	0.55	2.25	30	Good
Alternative *a*_4_	1	3	10	Fair
Global “worst” ( zj^)	0	0	0	None
Global “best” ( zj^^)	1	3	100	Excellent
Local “worst” ( $z_{j}^{*}$; *z*^#^)	0	0.9	10	Poor
Local “best” or “ideal” ( $z_{j}^{**}$; z^##^)	1	3	75	Excellent
$\left|z_{j}^{*}-Z_{j}^{**}\right|$	1	2.1	65	
$\sum_{i=1}^{m}z_{j}(a_{i})$	2.29	8.4	130	

*Notes*: We assume *c*_4_ categories correspond to numbers (None = 0, Poor = 25, Fair = 50, Good = 75, Excellent = 100); we assume local “worst” and “best” “worst” and “ideal” values, respectively, for compromise programming.

**Table 3:** Global scaling calculations for the hypothetical example. All values rounded to nearest whole number.

	Criterion *c*_1_	Criterion *c*_2_	Criterion *c*_3_	Criterion *c*_4_	*B_i_*	Rank
Alternative *a*_1_	0	75	75	100	63	2
Alternative *a*_2_	74	30	15	25	36	4
Alternative *a*_3_	55	75	30	75	59	3
Alternative *a*_4_	100	100	10	50	65	1

*Notes*: Linear transformation using Eq. (2) performed on *c*_1_ : *c*_4_ values to transform data on 0–100 scale.

**Table 4:** Local scaling calculations for the hypothetical example. All values rounded to nearest whole number.

	Criterion *c*_1_	Criterion *c*_2_	Criterion *c*_3_	Criterion *c*_4_	*B_i_*	Rank
Alternative *a*_1_	0	64	100	100	66	1
Alternative *a*_2_	74	0	8	0	20	4
Alternative *a*_3_	55	64	31	67	54	3
Alternative *a*_4_	100	100	0	33	58	2

*Notes*: Linear transformation using Eq. (3) performed on *c*_1_ : *c*_4_ values to transform data on 0–100 scale.

**Table 5:** Analytic hierarchy process calculations for the hypothetical example. All values rounded to nearest hundredth.

	Criterion *c*_1_	Criterion *c*_2_	Criterion *c*_3_	Criterion *c*_4_	*B_i_*	Rank
Alternative *a*_1_	0	0.27	0.58	0.56	0.35	1
Alternative *a*_2_	0.32	0.11	0.12	0.06	0.15	3
Alternative *a*_3_	0.24	0.27	0.23	0.26	0.25	2
Alternative *a*_4_	0.44	0.36	0.08	0.12	0.25	2

*Notes*: Vector normalization using Eq. (4) performed on *c*_1_ : *c*_3_ values; Saaty’s 9-point pairwise importance scale (Table 1) and eigenvalue analysis using Eqs. (5) and (6) performed on *c*_4_ values (see Supplementary material).

**Table 6:** Compromise programming calculations for the hypothetical example. All values rounded to nearest hundredth.

	Criterion *c*_1_	Criterion *c*_2_	Criterion *c*_3_	Criterion *c*_4_	*D_i_*	Rank
Alternative *a*_1_	1	0.13	0	0	0.08	2
Alternative *a*_2_	0.07	1	0.85	1	0.18	4
Alternative *a*_3_	0.20	0.13	0.48	0.11	0.06	1
Alternative *a*_4_	0	0	1	0.44	0.09	3

*Notes*: Assumed distance norm *p* = 2 in Eq. (7); lower values for *D_i_* correspond to preferred alternatives.

**Table 7:** Ecosystem service criteria, relative importance weights, and indicator data of four land management alternatives in Kgalagdi District, southern Botswana (adapted from Favretto et al., 2016).

Ecosystem service criterion (initial weight)	Indicator	Communal livestock grazing	Private cattle ranches	Private game ranches	Wildlife management areas	Range
Commercial food (0.17)	Max. net profit of meat production (US $/ha/yr)	0.64	1.21	−2.07	0	(−7.89,3.75)
	Min. stocking level (Ha/LSU)	11	14	9.5	160	(7,200)
Wild food (0.12)	Max. gathering of veld products	High	Low	Low	Medium	(Very low, Very high)
	Max. subsistence hunting	High	Very low	Very low	High^a^	(Very low, Very high)
Fuel (0.11)	Max. firewood collection	Very high	Medium	Medium	High	(Very low, Very high)
Construction material (0.10)	Max. collection of thatching grass and poles for fencing	Very high	Medium	Low	High	(Very low, Very high)
Groundwater (0.18)	Max. value of water extracted (US $/ha/yr)	0.84	0.97	0.15	0	(0,1.71)
Plant and livestock diversity (0.15)	Max. species and genetic diversity between forage species	Low	Medium	High	Very high	(Very low, Very high)
	Max. genetic diversity between livestock breeds	Low	High	Very low	Low	(Very low, Very high)
Climate regulation (0.08)	Max. value of carbon sequestration (US $/ha/yr)	1.7	1.7	1.3	0.3	(0,2.5)
Recreation (0.06)	Max. revenues from CBNRM trophy hunting and photographic safari (US $/hr/yr)	0	0	0	0.04	(0,0.09)
	Max. ecotourism potential	Low	Very low	High	Very high	(Very low, Very high)
	Max. wild animals diversity	Medium	Very low	Very high	Very high	(Very low, Very high)
Cultural/Spiritual benefits (0.03)	Max. presence of landscape features or species with cultural/spiritual benefits	Very high	Very low	Medium	Very high	(Very low, Very high)

a This value was incorrectly published as “Very high” in Favretto et al. (2016) (N. Favretto, personal communication).

**Table 8:** Results of MCDA analysis using Favretto et al. (2016) data set.

Iteration	Management alternative	Rank			
		Global scaling (Favretto et al., 2016)	Local scaling	Analytic hierarchy process	Compromise programming
Equal weights	Communal livestock grazing	1	1	2	2
	Private cattle ranches	4	3	3	4
	Private game ranches	3	4	4	3
	Wildlife management areas	2	2	1	1
Initial weighting (Fig. 2 in Favretto et al., 2016)	Communal livestock grazing	1	1	1	1
	Private cattle ranches	2	2	2	2
	Private game ranches	4	4	4	4
	Wildlife management areas	3	3	3	3
Sensitivity iteration 1 (Fig. 3a in Favretto et al., 2016)	Communal livestock grazing	1	1	1	2
	Private cattle ranches	2	2	2	1
	Private game ranches	4	4	4	4
	Wildlife management areas	3	3	3	3
Sensitivity iteration 2 (Fig. 3b in Favretto et al., 2016)	Communal livestock grazing	1	1	1	1
	Private cattle ranches	3	2	3	2
	Private game ranches	4	4	4	4
	Wildlife management areas	2	3	2	3
Sensitivity iteration 3 (Fig. 3c in Favretto et al., 2016)	Communal livestock grazing	1	1	1	1
	Private cattle ranches	3	2	3	3
	Private game ranches	4	4	4	4
	Wildlife management areas	2	3	2	2
Sensitivity iteration 4 (Fig. 3d in Favretto et al., 2016)	Communal livestock grazing	1	1	1	1
	Private cattle ranches	3	2	2	2
	Private game ranches	4	4	4	4
	Wildlife management areas	2	3	3	3
Sensitivity iteration 5 (Fig. 3e in Favretto et al., 2016)	Communal livestock grazing	1	1	1	1
	Private cattle ranches	3	2	3	3
	Private game ranches	4	4	4	4
	Wildlife management areas	2	3	2	2

*Note*: The sensitivity iterations are described in Favretto et al. (2016) and the specifics are not relevant to this current study.
